# A Retrospective Comparison of Narrowband-UVB Phototherapy in Pediatric Versus Adult Vitiligo

**DOI:** 10.3390/children12040466

**Published:** 2025-04-04

**Authors:** Kristin A. Tissera, Elena B. Hawryluk, Anna Cristina Garza-Mayers

**Affiliations:** 1 School of Medicine, Duke University, Durham, NC 27710, USA; 2 Department of Dermatology, Massachusetts General Hospital, Boston, MA 02114, USA; 3 Department of Immunology, Dermatology Section, Boston Children’s Hospital, Boston, MA 02115, USA; 4 Division of Dermatology, Seattle Children’s Hospital, Seattle, WA 98105, USA

**Keywords:** vitiligo, phototherapy, narrowband-UVB, pediatric phototherapy, pediatric

## Abstract

Background/Objectives: Vitiligo is an autoimmune condition causing melanocyte destruction and skin depigmentation. First-line treatments for vitiligo include topical medications and phototherapy; however, access and utilization of these treatments vary, particularly in pediatric patients. This study evaluates nbUVB use in pediatric versus adult vitiligo patients to better understand utilization in the pediatric population. Methods: A retrospective chart review study was conducted, collecting demographics and treatment characteristics for 102 adults and 19 children with vitiligo treated with nbUVB phototherapy at one institution. Statistical analysis included comparisons for categorical variables made using Chi-squared test or Fisher’s exact test, as appropriate, and using a non-parametric Mann–Whitney U test for continuous variables. Results: On average, adults underwent nbUVB for 23.8 months (range 0.5–418, median 9), while children had an average duration of 14.8 months (range 2–60, median 8). The average number of nbUVB sessions for adults was 83.8, whereas children had an average of 33.5 sessions. Overall, 59.6% of adults and 60.0% of children experienced repigmentation with nbUVB. Conclusions: Retrospective analysis indicated that the duration and frequency of nbUVB sessions varied widely among both adults and children. While the average duration of treatment was comparable between the groups, children underwent fewer sessions on average. This may reflect differences in physician recommendation, scheduling constraints, or treatment adherence. Despite this variability, most pediatric patients exhibited repigmentation, supporting nbUVB efficacy. Our results suggest nbUVB is underutilized in pediatric vitiligo. Addressing obstacles to access is crucial for improving treatment outcomes and quality of life.

## 1. Introduction

Vitiligo is characterized by depigmentation of the skin caused by the autoimmune destruction of melanocytes. Expert guidelines recommend narrowband-UVB phototherapy (nbUVB) in combination with topical therapy as first-line in the management of active non-segmental vitiligo in adults [1,2,3]. NbUVB induces pigmentation by stimulating the migration of melanocytes in the hair follicle sheath to the epidermis [4]. Phototherapy and topical therapy are thought to work synergistically to enhance repigmentation [5].

Evidence suggests that vitiligo is undertreated in both adults and children, with one study showing that 56.5% of their cohort of 13,449 patients did not receive any treatment during the first year following diagnosis [6]. The existing literature suggests that nbUVB is safe and effective in children, with few adverse effects, and no increased risk of skin cancer in adult vitiligo patients [4,7,8,9,10]. Nevertheless, topical corticosteroids and topical calcineurin inhibitors are most often prescribed by pediatric dermatologists [11]. In one study, only 38.9% of pediatric vitiligo patients were treated with nbUVB [7].

In this study, we retrospectively analyzed pediatric and adult patients treated at one institution with nbUVB, as a first step in characterizing utilization in pediatric vitiligo.

## 2. Materials and Methods

An IRB-exempt retrospective chart review was performed by searching the Mass General Brigham (MGB) Research Patient Data Repository (RPDR) for pediatric and adult patients with vitiligo and extracting clinical data. The primary outcome measure was the percentage of patients with documented nbUVB use. Statistical analysis included frequencies and proportions. Comparisons for categorical variables were made using a Chi-squared test or Fisher’s exact test, as appropriate, and using a non-parametric Mann–Whitney U test for continuous variables. *p*-values < 0.05 were considered statistically significant.

## 3. Results

As of December 2023, the total population of patients with vitiligo at MGB was 10,521 adults and 1909 children. We identified 102 adults and 19 children with vitiligo treated with nbUVB (Table 1). Non-segmental vitiligo was the most common type observed in 98.0% of adults (100/102), and in 42.1% of children (8/19) (Table 2). The face, including ears, emerged as the most frequently affected area, followed by arms and hands, in both age groups. Children with vitiligo who were treated with nbUVB had an average age of 9.92 years (range 5–13 years).

Most subjects, 92.2% of adults (94/102) and 100% of children (19/19), had undergone treatment prior to commencing nbUVB (Table 1). Among adults, 85.3% (87/102) had utilized topical corticosteroids (TCS), followed by 77.5% (79/102) using topical calcineurin inhibitors (TCI). In comparison, among children, 84.2% (16/19) had used TCS, with a slightly higher percentage, at 89.5% (17/19), employing TCI. Other prior therapies included topical and oral JAK inhibitors, oral corticosteroids, outdoor sun exposure, and prior and/or concurrent excimer laser or nbUVB at another institution.

During nbUVB, concurrent therapies were commonly used, with TCS used in 56.9% (58/102) and TCI used in 56.9% (58/102) of adults and TCS used in 42.1% (8/19) and TCI used in 84.2% (16/19) of children. Additionally, five adults and three children used topical JAK inhibitors. One adult patient used an oral JAK inhibitor alongside nbUVB.

The average duration of nbUVB for adults was 23.8 months (range 0.5–418, median 9). Among children, the average duration was 14.8 months (range 2–60, median 8). The most common treatment frequency recorded was 2–3 times a week. Adults received an average of 95.9 nbUVB sessions (range 1–597, median 46) while children underwent an average of 53.1 sessions (range 2–238, median 36.5). There was no statistically significant difference in the duration of treatment (*p* = 0.2246) and number of treatment sessions (*p* = 0.0631) documented for adults versus children, though both showed a decreased trend in children.

Among adults, 52.0% (53/102) of patients experienced repigmentation, while 33.3% (34/102) showed no change, and 14.7% (15/102) continued to experience depigmentation. In comparison, among 15 children, 60% (9/15) experienced repigmentation, 40% (6/15) showed no change, and none reported continued depigmentation. The remaining four pediatric patients did not have recorded clinical outcomes after treatment. Of note, pigment outcomes were not mutually exclusive. There was no statistically significant difference in the proportion of patients experiencing repigmentation (*p* = 0.3588).

Most patients experienced no side effects of nbUVB, with only one adult patient stopping therapy due to an adverse effect (Table 2). In adults, 9.80% (10/102) of patients reported symptoms of pruritus/irritation (1), burning sensation (8), and prolonged erythema (4). Only 3/19 children reported side effects: pruritus/irritation (3), burning sensation (3), prolonged erythema (2), and sunburn with blistering (1).

Phototherapy was ultimately discontinued in 75.5% of adults (77/102) and 78.9% of children (15/19). The reasons cited in the pediatric group included achieving a sufficient response in 5.26% (*n* = 1), lack of response in 31.6% (*n* = 6), difficulty in keeping appointments in 26.3% (*n* = 5), and other reasons such as the COVID-19 pandemic in 10.5% (*n* = 2).

## 4. Discussion

Our findings suggest that nbUVB is not regularly used at our institution in either pediatric or adult vitiligo patients. Our database search identified less than 1% of the MGB vitiligo patient population as receiving this treatment modality. Our cohort included 102 adults, 0.97% of the total population and 19 children, 0.99% of the total population. Duration and frequency of nbUVB sessions varied widely among both adults and children, with children undergoing fewer sessions and shorter treatment durations on average though without statistically significant difference. This may reflect differences in physician recommendation, scheduling constraints, or treatment adherence. Expert guidelines recommend 30–48 sessions prior to assessing treatment response, suggesting most of the children in our study were undertreated [3]. Despite this, most pediatric patients in the treated cohort exhibited repigmentation, despite having significantly more patients with segmental vitiligo, suggesting nbUVB efficacy in this population [7,9,12]. Ultimately, this suggests the need for further research in the pediatric population.

A recently published survey study of patients and caregivers evaluating access to and use of nbUVB to treat pediatric vitiligo revealed major barriers to accessing nbUVB, including it not being offered by physicians, difficulty attending appointments, and satisfaction with the current skin condition [13]. Despite high interest in nbUVB treatment among those surveyed, additional barriers to the use of nbUVB included challenges with insurance coverage, cost, and physical access to nbUVB treatment centers [13]. For patients experiencing barriers in access to nbUVB treatment, in our clinical practice we often encourage natural sunlight exposure or “heliotherapy” during summer months to provide some benefit.

A separate survey of pediatric dermatologists identified topical calcineurin inhibitors and topical corticosteroids as preferred vitiligo treatments, while nbUVB and excimer laser were reported to be less frequently used [11]. Our data support this in both children and adults. Additional studies on phototherapy implementation in both adults and children would allow for clearer guidelines and shared decision-making between families and providers. For instance, at our institution patients under age 7 are generally not treated due to concerns for tolerability, though there are no data to support a strict age cut-off. In an evolving treatment landscape, biologic treatments like JAK inhibitors in the treatment of inflammatory and autoimmune conditions like vitiligo are becoming more commonly used since the FDA approval of topical ruxolitinib (Opzelura) in July 2022 [14]. With this in mind, the role of nbUVB in the treatment of pediatric vitiligo requires further investigation, with regard to both efficacy and safety. Three of the pediatric patients in our study were on a topical JAK inhibitor concurrent with nbUVB treatment. Given the growing body of research demonstrating the effectiveness of topical JAK inhibitors, particularly in combination with phototherapy, future research could evaluate the benefit of dual treatment with topical JAK inhibitors and nbUVB [14].

Limitations of our analysis include its retrospective nature, relying on provider documentation, including reporting of repigmentation and depigmentation concurrently, limited reporting of body surface area, particularly in adult patients, and the absence of skin type reporting. In line with growing evidence, it is these authors’ belief that the most utilized skin color scale is limited in its accuracy and utility [15]. Nevertheless, it is possible that the shade of skin color affects repigmentation and response to nbUVB as well as patient and provider motivation to seek treatment. Our analysis was also limited to nbUVB performed at our institution, which does not include excimer laser and does not include home phototherapy units. Finally, our study is limited in its small size of patients with documented nbUVB use; at the same time, this illustrates the potential gap in access we hope to address.

## 5. Conclusions

Our study highlights that despite successful repigmentation with nbUVB phototherapy amongst vitiligo patients, there remains low utilization, which may be attributed to a variety of patient, disease, and healthcare system factors. As novel therapies for vitiligo emerge, it is important to ensure equitable access, including for pediatric patients [16,17]. Future research includes the identification and mitigation of barriers to use, potentially including expanding access to home phototherapy units. Collaborative efforts among healthcare providers, policymakers, and patient advocacy groups are necessary to decrease access disparity.

## Figures and Tables

**Table 1:** Demographics, vitiligo characteristics, and treatment characteristics of adult and pediatric patients with vitiligo treated with phototherapy by retrospective study.

Sex Assigned at Birth	Adults (*n* = 102)	Children (*n* = 19)	*p*-Value
Male	49 (48.0%)	7 (36.8%)	0.5169
Female	53 (52.0%)	12 (63.2%)	
Race/Ethnicity			
American Indian or Alaska Native	0 (0%)	0 (0%)	0.2580
Asian	15 (14.7%)	3 (15.8%)	
Black or African American	9 (8.8%)	4 (21.1%)	
Hispanic or Latino/a/x	37 (36.3%)	3 (15.8%)	
Native Hawaiian or Other Pacific Islander	0 (0%)	0 (0%)	
White	39 (38.2%)	9 (47.4%)	
Other/Not Available	11 (10.8%)	3 (15.8%)	
Type of Vitiligo			
Nonsegmental	100 (98.0%)	8 (42.1%)	<0.0001
Segmental	2 (1.96%)	6 (31.6%)	
Other/Not Available	0 (0%)	5 (26.3%)	
Involved Areas			
Face including ears	63 (61.8%)	16 (84.2%)	0.1043
Scalp	10 (9.8%)	3 (15.8%)	0.7113
Neck	24 (23.5%)	4 (21.1%)	0.9999
Chest	29 (28.4%)	1 (5.3%)	0.0632
Abdomen	25 (24.5%)	4 (21.1%)	0.9749
Back	32 (31.4%)	4 (21.1%)	0.5286
Arms	43 (42.2%)	4 (21.1%)	0.1398
Axilla	19 (18.6%)	2 (10.5%)	0.5988
Wrists	12 (11.8%)	4 (21.1%)	0.4663
Hands including fingers	57 (55.9%)	3 (15.8%)	0.0031
Suprapubic skin	4 (3.9%)	0 (0%)	0.8579
Genitals	22 (21.6%)	1 (5.3%)	0.1787
Inguinal Folds	11 (10.8%)	2 (10.5%)	0.9999
Buttocks	6 (5.9%)	1 (5.3%)	0.9999
Legs	41 (40.2%)	9 (47.4%)	0.7420
Ankles	9 (8.8%)	2 (10.5%)	0.9999
Feet including toes	30 (29.4%)	1 (5.3%)	0.0539
%BSA			
<1	5 (5.0%)	2 (10.5%)	0.0015
1–5	5 (5.0%)	8 (42.1%)	
5–10	9 (8.9%)	1 (5.3%)	
10–25	9 (8.9%)	1 (5.3%)	
25–50	4 (4.0%)	1 (5.3%)	
50–75	1 (1.0%)	0 (0%)	
>75	2 (2.0%)	0 (0%)	
Not Available	66 (65.3%)	6 (31.6%)	
Treatment prior to nbUVB			
Yes	94 (92.2%)	19 (100%)	0.4470
No	8 (7.8%)	0 (0%)	
Prior topical corticosteroid use			
Yes	87 (85.3%)	16 (84.2%)	0.9999
No	15 (14.7%)	3 (15.8%)	
Prior topical calcineurin inhibitor use			
Yes	79 (77.5%)	17 (89.5%)	0.3790
No	23 (22.5%)	2 (10.5%)	

**Table 2:** Retrospective analysis of nbUVB phototherapy in adults and children.

Duration of nbUVB Phototherapy (Months)	Adults (*n* = 102)	Children (*n* = 19)	*p*-Value
Mean [Range]	23.8 [0.50–418]	14.8 [2–60]	0.2246
Frequency of nbUVB phototherapy			
Once a week	4 (3.92%)	2 (10.5%)	0.5509
Twice a week	36 (35.3%)	6 (31.6%)	
Three times a week	57 (55.9%)	10 (52.6%)	
Not specified	12 (11.8%)	1 (5.26%)	
Total nbUVB phototherapy sessions			
Mean [Range]	95.9 [1–597]	53.1 [2–238]	0.0631
Vitiligo response to nbUVB			
Repigmentation	53 (52.0%)	9 (60.0%)	0.3588
No change	34 (33.3%)	6 (40.0%)	
Continued depigmentation	15 (14.7%)	0 (0.0%)	
Side effects			
Yes	10 (9.80%)	3 (17.6%)	0.7413
No	81 (79.4%)	14 (82.4%)	
Not reported	11 (10.8%)	2 (10.5%)	
If yes, side effect details			
Itchiness/irritation	1 (0.98%)	3 (15.8%)	0.0089
Burning sensation	8 (7.84%)	3 (15.8%)	0.5018
Pink erythema (<1 day)	4 (3.92%)	2 (10.5%)	0.5208
Sunburn with blistering	0 (0%)	1 (5.26%)	0.3438
Scarring	0 (0%)	1 (5.26%)	0.3438
nbUVB stopped due to side effect?			
Yes	1 (0.98%)	0 (0%)	0.9999
No	77 (75.5%)	15 (78.9%)	
Not reported/not stopped	24 (23.5%)	4 (21.0%)	
Reason for stopping phototherapy			
Sufficient response	11 (10.8%)	1 (5.26%)	0.7480
No response	14 (13.7%)	6 (31.6%)	0.1125
Too difficult to keep appointments	19 (18.6%)	5 (26.3%)	0.6467
Other: pandemic, side effect, not specified	26 (25.5%)	2 (10.5%)	0.2611
Adverse effects			
Pruritis/irritation	1 (0.98%)	3 (15.8%)	0.0089
Burning sensation	8 (7.8%)	3 (15.8%)	0.5018
Prolonged erythema	4 (3.9%)	2 (10.5%)	0.5208
Sunburn with blistering	0 (0%)	1 (5.26%)	0.3438

## Data Availability

Data are contained within the article.

## Abbreviations

The following abbreviations are used in this manuscript:

nbUVB	Narrowband-UVB phototherapy
MGB	Mass General Brigham
TCI	Topical Calcineurin Inhibitor
TCS	Topical Corticosteroid
